# Design of Asymmetric Nanofibers-Membranes Based on Polyvinyl Alcohol and Wool-Keratin for Wound Healing Applications

**DOI:** 10.3390/jfb12040076

**Published:** 2021-12-20

**Authors:** Diego O. Sanchez Ramirez, Iriczalli Cruz-Maya, Claudia Vineis, Cinzia Tonetti, Alessio Varesano, Vincenzo Guarino

**Affiliations:** 1 CNR-STIIMA (National Research Council-Institute of Intelligent Industrial Technologies and Systems for Advanced Manufacturing), Corso Giuseppe Pella 16, 13900 Biella, Italy; claudia.vineis@stiima.cnr.it (C.V.); cinzia.tonetti@stiima.cnr.it (C.T.); alessio.varesano@stiima.cnr.it (A.V.); 2 CNR-IPCB (National Research Council-Institute for Polymers, Composites and Biomaterials), Mostra d’Oltremare, Pad. 20, V.le J.F. Kennedy 54, 80125 Napoli, Italy; cdiriczalli@gmail.com

**Keywords:** wool-keratin, polyvinylalcohol, electrospinning, bilayered fibers, wound healing, asymmetric membranes

## Abstract

The development of asymmetric membranes—i.e., matching two fibrous layers with selected composition and morphological properties to mimic both the epidermis and dermis—currently represents one of the most promising strategies to support skin regeneration during the wound healing process. Herein, a new asymmetric platform fabricated by a sequential electrospinning process was investigated. The top layer comprises cross-linked polyvinylalcohol (PVA) nanofibers (NFs)—from water solution—to replicate the epidermis’s chemical stability and wettability features. Otherwise, the bottom layer is fabricated by integrating PVA with wool-keratin extracted via sulfitolysis. This protein is a biocompatibility polymer with excellent properties for dermis-like structures. Morphological characterization via SEM supported by image analysis showed that the asymmetric membrane exhibited average fiber size—max frequency diameter 450 nm, range 1.40 μm—and porosity suitable for the healing process. FTIR-spectrums confirmed the presence of keratin in the bottom layer and variations of keratin-secondary structures. Compared with pure PVA-NFs, keratin/PVA-NFs showed a significant improvement in cell adhesion in in vitro tests. In perspective, these asymmetric membranes could be promisingly used to confine active species (i.e., antioxidants, antimicrobials) to the bottom layer to support specific cell activities (i.e., proliferation, differentiation) in wound healing applications.

## 1. Introduction

Skin is the largest organ in the human body, which acts as a natural barrier to protect other tissues and organs, simultaneously regulating hydration and temperature [1]. The disruption of this tissue by wounds (e.g., surgical procedures, physical or chemical trauma, infection, or specific diseases) gives rise to a complex healing process constituted by hemostasis, inflammation, proliferation, and remodeling phases [2]. However, this reparation process in adults involves fibrosis, resulting in a scar more fragile than the original skin owing to the disorganized extracellular matrix (ECM) [3]. Thus, the development of biomaterials with tunable properties is necessary to support all the different phases of the wound healing process in terms of biocompatibility, hydrophilicity, degradation rate, morphology, mechanical strength, and active agent release [4].

PVA is a synthetic water-soluble polymer, widely used in biomedical and pharmaceutical applications because of its biocompatibility, biodegradability, and non-toxic properties. The presence of hydroxyl groups on PVA increases its hydrophilicity and capacity to create good physical/chemical interaction with other molecules [5]. Moreover, PVA has been broadly used in sponges [6], films [7], and hydrogels [8] for wound healing in combination with other natural or synthetic polymers such as alginate [9], chitosan [10], and PLA [11].

In the last years, there has been a substantial increase in studies focused on the fabrication of PVA-NFs as wound dressing devices owing to the high reproducibility of the electrospinning process that can be variously adapted to a wide range of polymers [12]. In particular, the integration of PVA with natural polymers such as proteins was adopted to enhance polypeptide processability and provide specific moieties—i.e., cell-binding sites and biomolecular signatures—able to mimic the innate capability of extracellular matrix to support cell interaction [13,14]. Besides, the relevance of proteins as an instructive biomaterial has been widely reported in the literature [15]. In particular, wool and hair keratins include cell-binding motifs, such as leucine-aspartic acid-valine (LDV) binding residues that allow supporting cell adhesion efficiently [16]. Accordingly, other studies have highlighted that keratin presents a strong hydrophilic behavior that improves human mesenchymal stem cells’ (hMSCs) proliferation in comparison with other proteins, like zein and gelatin [16]. In this context, the development of nanostructured membranes with asymmetric properties—owing to the addition of keratin to the fibers—certainly suggests new insights to design bioinspired platforms for interface applications.

This work proposes the design of double-layered devices for wound healing applications made of PVA-NFs and keratin/PVA-NFs. Morphological, wettability, and micro and macrostructural properties will be investigated and correlated with keratin/PVA blend ratios and post-treatment conditions (temperature). Lastly, the biological response of hMSC onto the different layers will be assessed by in vitro tests in order to validate their use in the fabrication of promising bi-layered platforms for wound healing applications.

## 2. Materials and Methods

### 2.1. Nanofiber Preparation

As reported in the literature [17], keratin (KS) powder was extracted by sulfitolysis from wool fibers. PVA (average Mw 130 kDa, CAS no. 9002-89-5) and all other reagents were acquired from Sigma-Aldrich (Milan, Italy), unless otherwise specified.

Solutions were prepared by dissolving separately KS in water at room temperature overnight and PVA in water at about 90 °C for 2 h, and then cooling them down over night. Two KS solutions were prepared at concentrations of 2.5 and 5.0% wt., and two PVA solutions were prepared at 9.0 and 15% wt. The PVA solution at 15% wt. was blended with KS solutions (2.5 and 5.0% wt.) at a weight ratio of 1:1 to have KSPVA1 and KSPVA2, respectively. The 9% wt. PVA solution was electrospun to obtain the pure PVA-NFs top layer.

**Figure 1:**
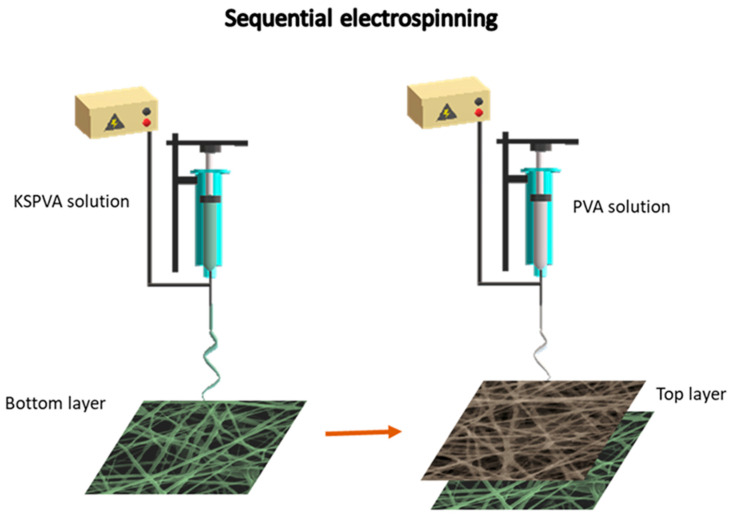
Scheme of sequential electrospinning for PVA and KSPVA nanofibers.

The production of nanofibrous-membranes was carried out using polymeric solutions in a home-made assembled single jet electrospinning (STIIMA-CNR, Biella, Italy), equipped with an SL50 high-voltage generator (SPELLMAN, Pulborough, UK), a KDS 200 high-precision syringe pump (KD Scientific Inc., Holliston, MA, USA), a stainless-steel needle (27 Ga), and a flat plate collector (Figure 1).

NFs were collected on aluminium using the process conditions reported in Table 1. All the fibrous samples were electrospun for 1 h at 24 °C and 60% RH. Lastly, they were thermally post-treated—at 155 °C for 3 min in air [18] or 180 °C for 2 h in air [19] in order to prevent the solubilization of proteins in water.

### 2.2. Morphological Characterization (SEM)

A scanning electron microscope (Zeiss EVO-10 GmbH—20 kV and 18 mm, Jena, Germany), was employed to study the morphology of membranes. NFs were coated with a gold-layer (20 nm) in argon (20 Pa) using a Quorum SC7620 Sputter Coater (20 mA for 120 s, Laughton, UK). The diameter distribution of NFs was measured on SEM images by the software Fiji-ImageJ 1.51.

### 2.3. Water Contact Angle (WCA)

The wettability of NFs was measured by an EasyDrop Standard Contact Angle Measuring system—DSA1 software (KRÜSS GmbH, Hamburg, Germany). The measurements were carried out six times for each sample at 20 °C and 65 ± 5% RH using 10 μL of ultrapure water (1.34 μS cm^−1^ and 72 mN m^−1^).

### 2.4. Water Uptake

In order to evaluate the water uptake, dry NFs were stored in a conditioned laboratory according to the moisture regain procedure. About 10 mg of thermally post-treated NFs was placed at 105 °C until a stable weight (dry state). The samples were stored at 20 ± 2 °C and 65 ± 5% relative humidity for 48 h and were weighted (wet state). The water uptake in percentage was calculated as the ratio between the weight difference from wet and dry states and the initial weight of NFs in dry state.

### 2.5. Fourier-Transform Infrared Spectroscopy Analysis (FTIR)

A spectrometer (Thermo Nicolet iN10, Madison, WI, USA) with an iZ10 module and a Smart Endurance accessory was used to record the spectrums in ATR between 4000 and 650 cm^−1^ (50 scans and 4 cm^−1^).

The secondary structure quantification of samples was carried out by fitting the Amide I peak with Gaussians. For all spectrums, a baseline correction was employed between Amide I and Amide II peaks. The wavenumber of each secondary structure was determined through the second derivate method using the quadratic Savitzky–Golay smoothing (third-order polynomial with five points). The frequency of secondary structures was assigned as follows: intermolecular β-sheets 1611, 1618, 1625, and 1695 cm^−1^ [20,21,22]; intramolecular β-sheets 1674 cm^−1^ [23]; the bands between 1630 and 1640 cm^−1^ assigned to inter and/or intramolecular β-sheets [23,24], and called “β-sheets II”; β-turns 1668, 1682, and 1689 cm^−1^ [21,22]; α-helix 1645, 1651, and 1659 cm^−1^ [25,26,27]; and random coil 1645, 1651, and 1659 cm^−1^ [25,26]. Furthermore, the small bands’ contributions due to side-chain (1594 and 1604 cm^−1^) and carboxylic groups adsorptions (1714, 1725, and 1731 cm^−1^) were also considered [25,28,29], but the quantification of secondary structures was carried out excluding those areas. Data were analyzed using the function peak analyzer of OriginLab-Pro 8.0. As reported in the literature, the fitting of Amide I peak was carried out limiting the values of full width at half maximum (FWHM) between 10 and 30 cm^−1^, allowing any positive value for the height of Gaussians and fixing the band position [25].

### 2.6. Cell Culture Tests

For in vitro assays, human mesenchymal stem cells (hMSCs, SCC034 from Sigma-Aldrich, Milan, Italy) from 4–6 passages were used. Firstly, hMSCs were cultured to reach the confluence in a 75 cm^2^ cell culture flask in Eagle’s alpha minimum essential medium (α-MEM) supplemented with 10% fetal bovine serum (Sigma-Aldrich, Milan, Italy), antibiotic solution (streptomycin 100 µg mL^−1^ and penicillin 100 U mL^−1^, Sigma-Aldrich), and 2 mM of L-glutamine, incubated at 37 °C in a humidified atmosphere with 5% CO_2_ and 95% air. Before in vitro studies, membranes were cut to be placed in a 96-well cell culture, washed and sterilized in 70% ethanol for 30 min, and then washed three times with phosphate buffered saline (PBS). All the studies were conducted three times by triplicate. To evaluate the influence on cell adhesion of KSPVA1 and KSPVA2 treated at different temperatures (180 and 155 °C), hMSCs were seeded onto round samples, at a 2 × 10^4^ for cell adhesion after 24 h. A cell culture plate was used as a control. The morphology of cells was observed at 24 h. Samples were rinsed three times with PBS to remove the non-attached cells, then attached cells were fixed with 4% paraformaldehyde. Samples were washed with PBS to remove the fixing agent, and then dehydrated in graded series of ethanol (25–100%) and air dried. The samples were sputter-coating and examined by FESEM under low-vacuum conditions (SEM) (Quanta200 FEI, Eindhoven, The Netherlands).

As for proliferation analyses, counting kit-8 assay (CCK-8; Dojindo Laboratories, Kumamoto, Japan) was performed. hMSCs were seeded at 1 × 10^4^ onto the different groups of KSPVA fibers and PVA. Briefly, the culture media was removed and changed at 1, 3, and 7 days by 100 µL of fresh medium with 10 µL of CCK-8 reagent per well. After 4 h of incubation, the supernatant was collected, and absorbance was measured at 450 nm using a microplate reader. The results are presented as mean ± standard deviation (*n* = 3). Analysis of variance (ANOVA) Tukey’s post hoc was used to detect differences between groups. A value of *p* < 0.05 was considered to determine statistically significant differences.

## 3. Results

**Figure 2:**
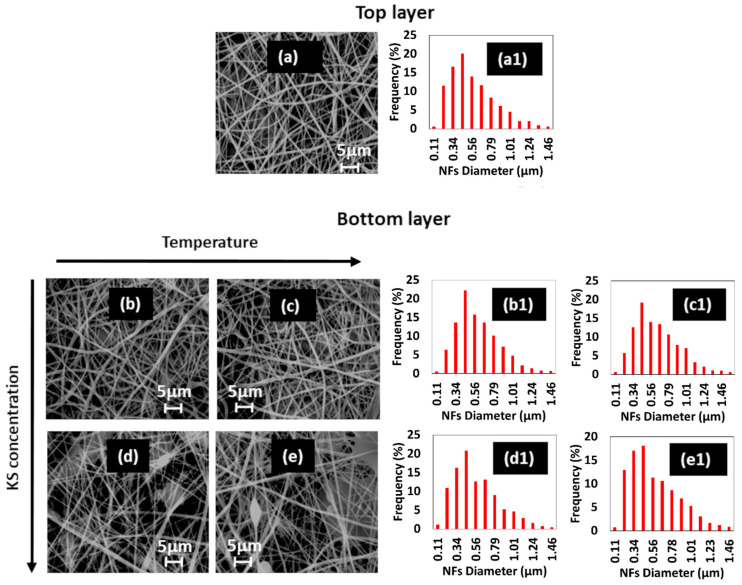
SEM images for samples: (**a**) PVA as spun, (**b**) KSPVA1—155 °C, (**c**) KSPVA1—180 °C, (**d**) KSPVA2—155 °C, and (**e**) KSPVA2—180 °C. The corresponding diameter distribution of NFs was labeled with number 1.

The morphology and diameter distribution of every single layer are reported in Figure 2. By the SEM images, it is possible to remark that neither thermal treatment (155 °C for 3 min and 180 °C for 2 h) nor a greater amount of KS considerably modified the diameter distribution of NFs (maximum frequency diameter 450 nm, range 1.40 µm). Different trends were recognized for the defects (i.e., beads) that increase with the amount of KS. In the literature [30,31], PVA shows a melting point and an onset for thermal degradation above 200 °C. Considering that, any effect of melt or thermal degradation of PVA on NFs can be excluded. Furthermore, it is necessary to highlight that both thermal treatments have different impacts on NFs. A short time at 155 °C might decrease the amorphous region of PVA and increase the crystalline region of PVA, hindering the diffusion of water molecules into the PVA structure [32]. Using a temperature of 180 °C for 2 h, not only could the crystalline structure of PVA rise in KSPVA, but also cross-linking reactions between carboxylic groups and amine residues of KS can occur [19].

The WCAs of all samples are reported in Table 2. Because the water drops were easily absorbed by NFs-membranes, the WCA was estimated at two times: t_i_ = 0 s and t_f_ = 3.3 s. Furthermore, the WCA variation over time (Δ) was estimated by the subtraction of WCA at t_i_ and t_f_. From Table 2, it can be observed that a greater temperature considerably reduced the wettability of NFs (WCA_ti_). At 180 °C, this effect was reinforced by the addition of KS. However, at 155 °C, the effect of KS on wettability was small, in particular for KSPVA2. On the other hand, the interaction between water and NFs over time was enhanced thanks to the presence of KS. However, this interaction was broadly minimized by the post-treatment at the greatest temperature. In fact, the smallest Δ was obtained for KSPVA1 at 180 °C—2 h, which could regulate the diffusion of aqueous fluids.

Water uptakes of dry NFs are listed in Table 3. Pure PVA NFs have the lowest water uptakes, close to ~3%. The water uptakes increase as the KS content increases. The maximum water uptakes were measured on KSPVA2 samples with values of 16.4% on the sample treated at 155 °C and 9.7% on the sample treated at 180 °C.

**Figure 3:**
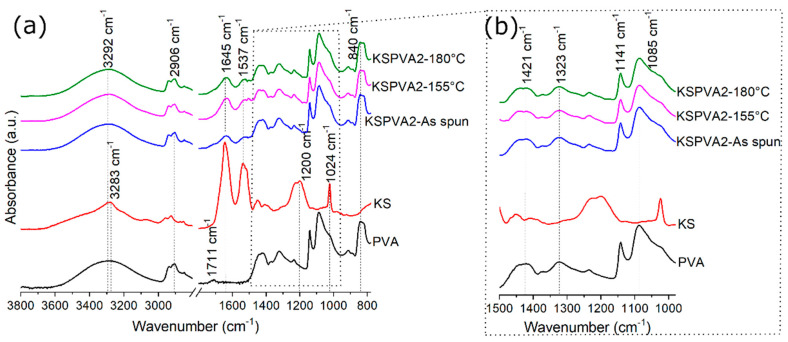
FTIR spectrums before and after post-treatment for NF-membrane made of KS and PVA in water—KS (lyophilized powder) and PVA (powder). (**a**) Spectrum from 3800 to 2800 cm^−1^ and from 1800 to 780 cm^−1^. (**b**) Spectrum from 1500 to 980 cm^−1^.

FTIR spectrums of all samples are reported in Figure 3 except for KSPVA1, whose spectrums were analogous to those reported by KSPVA2. As KS spectrum is concerned, Amide A peak at 3283 cm^−1^ is assigned to the stretching vibrations N–H bonds. Amide I peak at 1645 cm^−1^ and amide II peak at 1537 cm^−1^ are assigned to the stretching vibrations of C=O bonds and the in-plane bending modes of N–H bonds, with some contributions of C–N stretching vibrations. Amide III peak at 1200 cm^−1^ is assigned to an in-phase combination of N–H in-plane bending, C–N stretching vibrations, C–C stretching, and C=O bending vibrations [33], but it also depends on the nature of side-chain groups and hydrogen bonding [34]. The peak at 1024 cm^−1^ is related to the stretching vibration of the Bunte’s salt residues [35].

Regarding PVA, the peak at 3292 cm^−1^ is attributed to O–H stretching vibration, including free hydroxyl groups and inter/intra-molecular hydrogen bonds [32,36]. The side hydroxyl groups in PVA chains interact with each other and form strong hydrogen bonds [32]. The peak at 2906 cm^−1^ corresponds with the asymmetric and symmetric stretching of –CH_2_ groups [32,36]. The small peak at 1711 cm^−1^ is associated with the carbonyl group (C=O), which is caused by unhydrolyzed acetate groups and free acetate ions in PVA [32]. The two peaks at 1421 and 1323 cm^−1^ are assigned to the bending of C–H [32]. The peak at 1141 cm^−1^ is mainly ascribed to the crystallinity of PVA, associated with C–O in the crystalline region [32]. The peak at 1085 cm^−1^ corresponds to the C–O stretching of PVA [32,36]. The peak at 840 cm^−1^ is assigned to C–C vibrations [37].

In order to study the effect of KS and thermal treatment on the crystallinity of PVA inside KSPVA-NFs, the peak ratio between the bands at 1141 cm^−1^ and 1085 cm^−1^ was calculated, Table 4. In the literature [36], the importance of these peaks to characterize the crystallinity of PVA has already been demonstrated. As observed in Table 4, the electrospinning process has a negative effect on PVA-crystallinity, but at the same time, the use of KS has a positive impact on this property. In terms of thermal treatment, it is observed that a greater temperature increases crystallinity on pure PVA-NFs. The effect of simultaneous variations in KS content and temperature on PVA-crystallinity is at a maximum at 155 °C with 33% wt. KS and minimum at 180 °C with 17% wt. KS.

Table 5 reports the content of secondary structures for KS inside NFs-membranes, as solvents and polymers can alter the hydrogen bonds and the conformational properties of polypeptides [38,39,40].

Compared with pure-KS (lyophilized powder), the electrospinning of KS together with PVA seems to stabilize the structure’s α-helix, also reducing the content of random coil, β-sheet II, and intramolecular β-sheet conformations. In all cases, a rise in β-turn was observed, showing that the electrospinning process can influence this structure. Additionally, a more significant amount of KS on KSPVA-NFs increased the intermolecular β-sheet conformations (resulting in protein aggregation); this effect is increased by the thermal treatments. Table 5 indicates that the use of PVA has a positive effect on the α-helix structures of KS, which demonstrates the importance of employing a polymer capable of forming a great deal of hydrogen bonds. In terms of thermal treatment, samples with KS show an interesting result, as some changes on the secondary structures of KS are similar to those obtained for PVA-crystallinity inside NFs (Table 4). For example, when simultaneous variations of temperature and KS content are taken into account, a decrease in PVA-crystallinity corresponds with a reduction in α-helix structures. Similar behaviors are noticed on random coil, β-turn, and β-sheet II.

**Figure 4:**
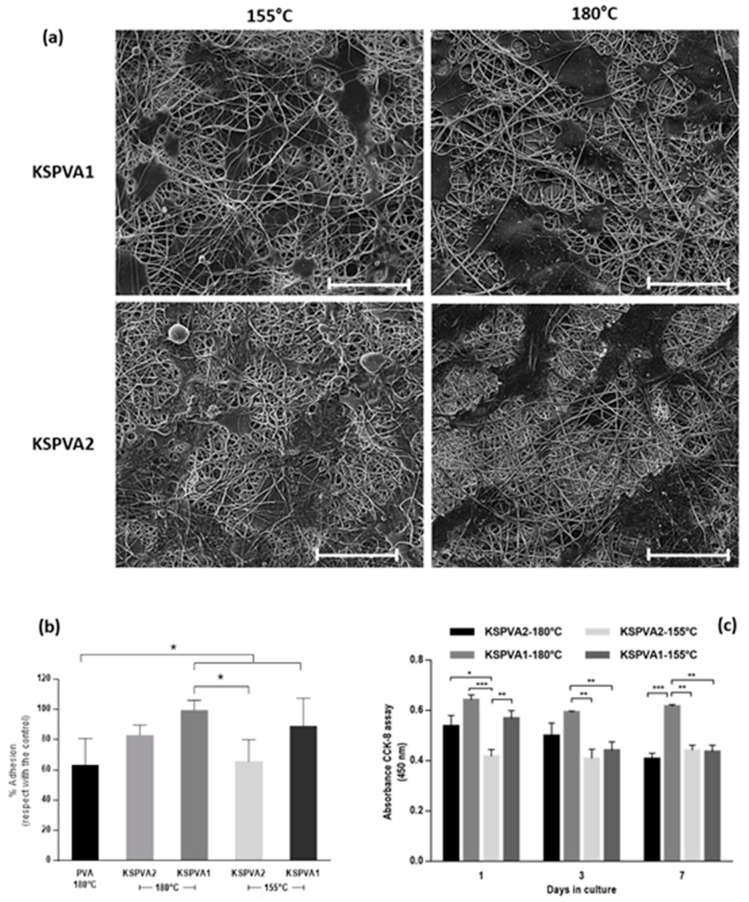
SEM images (scale bar 50 µm) (**a**) and quantitative results (**b**) of hMSCs’ adhesion onto KSPVA fibers after 24 h. (**c**) hMSCs’ viability assay on KSPVA2 and KSPVA1 treated at different temperatures. (* *p* < 0.05, ** *p* < 0.01, *** *p* < 0.001).

According to previous evidence, single NFs-layers—with and without keratin—have been tested by in vitro studies. Qualitative evaluation by SEM of hMSCs’ adhesion and spreading after 24 h onto different KSPVA samples are shown in Figure 4a. Quantitative adhesion results were calculated assuming that the total cell attachment corresponds to the cell culture plate. The average percentage of attached cells in all KSPVA-NFs overcame the value of 60%, better than PVA-NFs (Figure 4b). Cell adhesion after 24 h was around 90% in KSPVA1, independently of the temperature treatment. This result is ascribable to the presence of wool-keratin onto NFs (owing to the presence of integrin binding motifs [41]), as well as to the variations in WCA, PVA-crystallinity, and the secondary structure content of KS.

The hMSCs’ proliferation was measured by the absorbance of CCK-8 reagent, which is proportional to living cells (Figure 4c). Because the KSPVA1 sample at different temperature treatments showed better cell adhesion after 24 h, the hMSCs’ proliferation also reflected similar results. For the following days, cell proliferation values of KSPVA1 were almost constant for samples treated at 180 °C; meanwhile, the proliferation values of KSPVA1 treated at 155 °C decreased after 3 days. These results could be related to better stability of fibers conferred by the temperature treatment without altering the morphology and protein chains, as reported previously [19]. Moreover, the secondary structure content of KSPVA1—180 °C allows preserving more efficiently the biological stability of wool-KS, as confirmed by the biological results. It is noteworthy that no increase in cell proliferation was recorded in any other case, independently of temperature treatment and composition. This could be because of the effect of keratin release that negatively influences surface and mechanical properties of NFs-membrane, thus compromising cell viability after 24 h [42].

## 4. Discussion

Over time, the production of NFs by electrospinning has been recognized as an invaluable processing technique for the fabrication of substrates for skin substitution and wound healing [43]. Indeed, this technology joined the need to manufacture as nature by combining cells, ECM, and/or biomaterials to produce intricate living or non-living biological products. In the case of wound healing applications, it is well known that dressing materials play a crucial role during the repair processes in terms of surface protection, molecular transport, ion delivery, bacteriostatic function regulation, and cell interactions [44,45,46]. From this point of view, electrospun-NFs must not contain any traces of solvents post-fabrication—accordingly, the use of solvents such as TFE, DMF, or DCM is only restricted to pharmaceutical use by the U.S. Food and Drug Administration [47]. The polymer processing in aqueous solutions allows to minimize the manufacturing impact on the patient’s health, also facilitating the clinical translation of electrospinning products into green materials [48].

By and large, scaffolds must have functional architectures with tunable chemical and structural properties, supporting wound healing processes and simultaneously inhibiting bacteria activities [49,50]. However, most substrates for wound healing are still based on single-layer NFs, which partially satisfy these requirements. Currently, several efforts have been spent to design innovative strategies to increase the chemical and structural complexity of electrospun dressing platforms that overcome the limits of traditional dressings (i.e., cotton knitted textiles) without any active function in the healing process [51]. Other strategies also proposed the integration of nanoparticles loaded with antibiotics (i.e., such as tetracyclines [52] or other actives macromolecules (e.g., proteins [53] or essential oils [54])) with antibacterial properties to treat local infections accurately.

This work proposes an alternative approach that involves the use of electrospun asymmetric platforms as a strategy to replicate anatomic skin features during the repair process, with relevant enhancements in wound healing [55]. Asymmetry membranes are two-layered structures capable of mimicking the properties of both skin layers—epidermis and dermis. On the top (epidermis side), the system is characterized by a layer of cross-linked PVA-NFs that assure a dense structure with sufficient wettability to play a protective role of the wound site, limiting rapid dehydration of the surface and minimizing the diffusion of microorganisms (i.e., bacteria). On the bottom (dermis side), the system presents an interconnected porous layer with high absorption capacity to regulate the fluid adsorption, molecular transport, and complete drainage of the wound site. Indeed, the swelling properties of PVA-NFs are improved by wool-keratin with its bioactive properties that assure a more efficient interface with cells and enhanced intrinsic antibacterial properties, as reported in several studies [56].

In comparison with other epithelial components (i.e., gelatin, elastin, collagen, and sericin), extracted-keratin is a biodegradable and water-soluble protein with a cytocompatibility and non-immunogenic response due to their peculiar cell-binding motifs (i.e., arginine-glycine-aspartic acid) [57,58]. Recent studies confirmed the advantages of using keratin electrospun-NFs for wound healing applications because of their high porosity and capacity to absorb exudates [59]. Different content of KS and thermal treatments enables modifying the wettability, the microscopic properties of NFs, and the secondary structure content of KS, which confer different properties in terms of flexibility and chain elasticity, able to slightly influence cell response in vitro, in agreement with previous studies [60]. Accordingly, KS-NFs have also been combined with other proteins such as gelatin to design bilayered platforms able to improve the in vitro interactions with L929 fibroblasts, also promoting in vivo earlier vascularization and better skin wound healing [61]. In our work, we have proposed to use PVA, thanks to relevant benefits in terms of chemical stability and transport properties that characterize ionotropic polymer—such as PVA—with respect to structural proteins—such as gelatin.

Indeed, PVA and KS are recognized biocompatible materials, able to influence the cell-material interaction mechanisms in terms of adhesion and spreading, as well as assuring an accurate modulation of mechanical properties [62]. On the one hand, the combination of KS and PVA can improve the in vitro stability of the upper layer and, on the other hand, ensure an optimal mass transport of small molecules through the bottom one, giving, in perspective, the opportunity to more efficiently exchange the molecular signal at the wound site and to support the release of antimicrobial (i.e., antibiotic) molecules to fight bacteria through controlled release strategies.

## 5. Conclusions

This work proposes the design of electrospun-NFs from aqueous solutions to assembly asymmetric membranes as a promising strategy to support skin regeneration and wound healing. The top layer is composed of PVA-NFs to replicate the chemical stability and wettability features of the epidermis. Morphological studies confirmed that PVA-NFs satisfy ideal requirements for the top layer in terms of diameter distribution and porosity. Secondly, the bottom layer is fabricated by integrating PVA with wool-keratin extracted via sulfitolysis. In vitro tests highlighted an evident improvement of cell adhesion due to the presence of keratin and its recognized biocompatibility and wettability, suitable to mimic the properties of the dermis. Furthermore, the KS content and thermal treatment of NFs were demonstrated to have a relevant effect on PVA-crystallinity, whose variations influence the secondary structure content of proteins at different levels (i.e., α-helix and β-turns). The combination of PVA and high in vitro stability and bioactive proteins like keratin could be promising to design asymmetric membranes able to confine active species (i.e., antioxidants, antimicrobials) into the bottom layer and sustain, more specifically, cell activities (i.e., proliferation, differentiation) in the wound site.

## Figures and Tables

**Table 1:** Electrospinning conditions.

Samples	Solvent	Total Polymer Concentration (% wt)	Polymer Blend (% wt)	Flow Rate (mL min^−1^)	Tip Collector Distance (cm)	Voltage (kV)	Needle Inner Diameter (mm)
KSPVA1 (Bottom-layer)	Water	8.75	17/83 KS/PVA	0.015	30	25	0.4
KSPVA2 (Bottom-layer)	Water	10.00	33/67 KS/PVA	0.015	25	25	0.4
PVA (Up-layer)	Water	9.00	100 PVA	0.008	30	23	0.4

**Table 2:** WCA for all NFs-membranes at two different times.

Sample	Thermal Post-Treatment	WCA_ti_ (°), 0 s	WCA_tf_ (°), 3.3 s	Δ (°), WCA_ti_—WCA_tf_
PVA	155 °C—3 min	50.2 ± 2.4	36.6 ± 1.4	14
KSPVA1	155 °C—3 min	78.3 ± 4.0	36.7 ± 10.1	42
KSPVA2	155 °C—3 min	52.6 ± 3.3	18.3 ± 6.1	34
PVA	180 °C—2 h	67.4 ± 4.1	51.6 ± 5.3	16
KSPVA1	180 °C—2 h	131.0 ± 4.8	123.7 ± 8.7	7
KSPVA2	180 °C—2 h	127.8 ± 3.1	107.1 ± 6.1	21

**Table 3:** Water uptake percentages of electrospun nanofibers.

Sample	Thermal Post-Treatment	
	155 °C—3 min	180 °C—2 h
Pure PVA	3.4%	3.3%
KSPVA1	4.9%	8.5%
KSPVA2	16.4%	9.7%

**Table 4:** Peak ratio *I*_1141_/*I*_1085_.

Thermal Post-Treatment	Sample		
	PVA	KSPVA1	KSPVA2
As spun	0.5813	0.7405	0.7508
155 °C—3 min	0.6779	0.7547	0.7568
180 °C—2 h	0.7611	0.7244	0.7554

*I*_1141_/*I*_1085_ for PVA powder is 0.7562.

**Table 5:** Protein secondary structure content (%).

Sample	Intermolecular β-Sheet	Intramolecular β-Sheet	β-Sheet II	β-Turn	Random Coil	α-Helix
KS (lyophilized powder)	23.9	6.5	24.2	11.3	6.7	27.4
KSPVA1, as spun	23.2	5.5	13.4	17.2	2.8	37.9
KSPVA1, 155 °C—3 min	24.8	5.5	10.7	16.7	1.8	40.4
KSPVA1, 180 °C—2 h	25.8	5.6	14.1	18.5	6.0	30.0
KSPVA2, as spun	27.2	5.3	7.3	16.4	0.8	43.0
KSPVA2, 155 °C—3 min	27.0	5.1	13.4	13.3	1.7	39.5
KSPVA2, 180 °C—2 h	31.9	4.7	8.9	14.7	1.7	38.1

## Data Availability

Not applicable.
